# Interoceptive Basis to Craving

**DOI:** 10.1016/j.neuron.2007.03.024

**Published:** 2007-04-19

**Authors:** Marcus A. Gray, Hugo D. Critchley

**Affiliations:** 1 Brighton and Sussex Medical School, University of Sussex Falmer Campus, Brighton BN1 6PX, United Kingdom

## Abstract

Awareness of one's physiology is an important component of emotion. How might these processes be related to addiction? In a recent issue of *Science*, Naqvi et al. demonstrated that smoking addiction is disrupted by damage to the insula cortex. This suggests that brain circuits mediating interoception also contribute to craving states.

## Main Text

Addictions hijack brain systems controlling emotional and motivational behavior that are fundamental to adaptive survival. Feeling states, such as craving, are the experiential component of motivational behavior, representing consciously accessible expressions of motivational need. Primary feeling states represent an elegant means of maintaining physiological homeostasis; feelings such as hunger, cold, or thirst motivate nutrient absorption (facilitating thermoregulation and rehydration) and may be considered homeostatic emotions. Evolutionary development of emotional systems within social environments may explain a hierarchy of feelings, where more complex emotions can anticipate potential social challenges to homeostatic and internal integrity and contribute to mnemonic reinforcement of adaptive behaviors. Motivational stimuli such as emotive cues engender involuntary bodily responses (for example, in patterns of autonomic arousal). The interoceptive feedback of these dynamic bodily reactions directly influences feeling states and affective experience. This process is central to influential peripheral theories of emotion (e.g., James, 1894; Schachter and Singer, 1962). Correspondingly, the brain's representation of viscerosensory “markers” can guide cognitive and motivational behavior (Nauta, 1971; Damasio, 1994), and the activity of many brain regions implicated in cognition and effect are sensitive to changes in peripheral arousal (reviewed in Critchley [2005]). One recent formulation proposes that the conscious experience of anxiety arises from “prediction error” where the actual arousal state (conveyed interoceptively within insula cortex) is different from the anticipated/expected arousal state of the body (Paulus and Stein, 2006). Recent research by Naqvi and colleagues (2007) in *Science* provides strong evidence that interoceptive processes within insula cortex underlie craving associated with nicotine addiction.

The addictive nature of tobacco smoking is illustrated by the difficulty smokers experience in quitting, despite the fact that deaths attributable to tobacco smoking are predicted to reach 6.4 million per year by 2015 (Mathers and Loncar, 2006). Craving, typically experienced within acutely abstinent smokers on withdrawal, is understood to be a key component in the cycle of addiction. Naqvi and colleagues (2007) examined patients who were smokers before they suffered regional brain damage. They report smoking addiction is attenuated or abolished following focal damage to insula cortex. Almost half of the patients studied gave up smoking after their acquired brain injury. However, if the lesion involved insula cortex, the patient was more than twice as likely to stop smoking as patients with damage that did not affect insula cortex. There were also striking differences between patients in the experience of stopping smoking; almost all the patients with insula damage gave up without experiencing urges to smoke or difficulties in quitting. In contrast, over three quarters of patients who quit smoking after noninsula damage reported subjective difficulty, urges to smoke, and even occasional relapses, consistent with ongoing symptoms of smoking addiction. This work highlights the contribution of insula cortex to neural processes that maintain addictive behaviors and resistance to behavioral intervention. In the words of authors, “this result suggests that the insula is a critical neural substrate in the addiction to smoking.”

Addiction neuroscience has been valuably informed by animal models. Processes that characterize addiction, such as habit learning and reward signaling, are supported by dopaminergic mechanisms within striatal and prefrontal circuitry (Hyman et al., 2006). The neurobiological power of these behavioral models has perhaps contributed to underemphasis of the subjective dimension of addiction, where the conscious experience of urge and tension during withdrawal may be treated as an epiphenomenon. The observation of Naqvi and colleagues (2007) indicates the insula cortex is a likely substrate for conscious addictive urges associated with smoking addiction. What has this to do with interoception? Interoceptive models of emotion propose that subjective feelings ultimately arise from interoceptive signals. Feeling states including urge and anxiety are related. For example, the tension of addictive withdrawal, and relief on relapse, shares features with obsessive compulsive disorder, a psychiatric condition in which obsessions are anxiogenic and compulsions relieve that anxiety. A convergence of evidence from human neuroimaging, clinical studies, and basic science research has helped elucidate the neural architecture supporting control of bodily arousal states and representation of interoceptive feedback from the body. Until quite recently, these processes were largely ignored within human psychology and typically attributed to reflexive engagement of brainstem and hypothalamus. There is now recognition that insula cortex is important for the representation of viscerosensory information (Craig, 2005). Consistent with “interoceptive emotion,” the insula also plays a critical role in affective behavior, often in conjunction with orbitofrontal and anterior cingulate cortices. Anatomically, Craig (2002) has argued for distinct specialization of motivationally salient “interoceptive” lines of information, even in the periphery (this is an inclusive definition: sensual touch, pain, and thermal information from the skin are treated as interoceptive visceral sensations).

**Figure 1:**
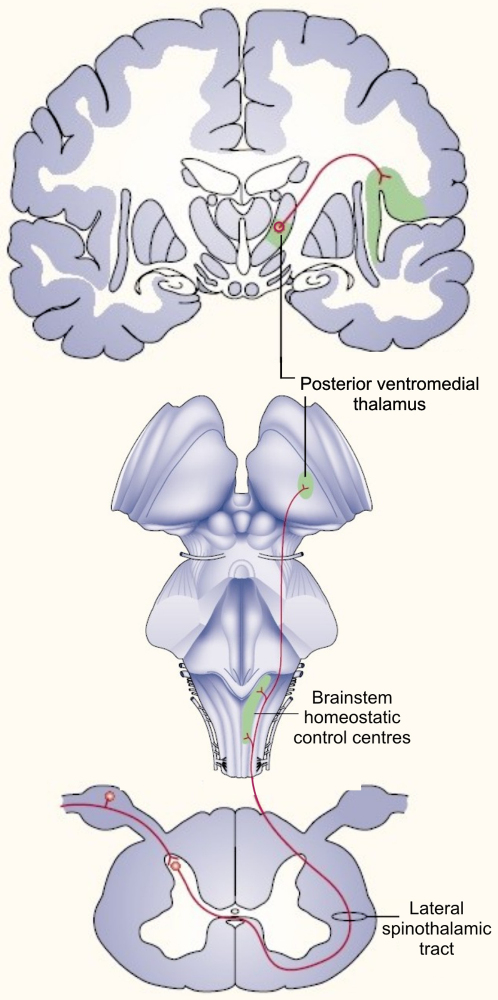
Lamina 1 Spinothalamocortical Pathway Small diameter primary afferents project homeostatic and viscerosensory information to the nucleus of the solitary tract (and in humans also directly to the thalamus). Projection fields within the dorsal posterior insula cortex present a cortical image of the bodies' physiology. Figure adapted by permission from Macmillan Publishers Ltd: *Nature Reviews Neuroscience* (Craig, 2002), copyright 2002.

Within the primate brain, interoceptive information relays via thalamus to insula cortex where sequential rerepresentations permit associative integration with other processing streams (Figure 1). In humans, neuroimaging evidence suggests the right anterior insula as a terminal node in interoceptive processing where there is declarative conscious access to visceral sensation as subjective feelings. Craig's evidence for the representation of feelings in anterior insula cortex is endorsed by many separate studies (including his own in relation to thermal sensation; across viscerosensory and emotional challenges, feeling states appear related to insula activity [particularly within right anterior insula]; reviewed in Craig [2002], Critchley [2005]). We suggest that the effect of insula lesions on smoking addiction is attributable to disruption of the capacity to represent (hence respond to) internal interoceptive signals that are perceived subjectively as anxiety and tension. These signals are generated in response to psychological processing of smoking-related cues generated by conditioned reinforces (perhaps more so than to the physiological effects of nicotine withdrawal).

**Figure 2:**
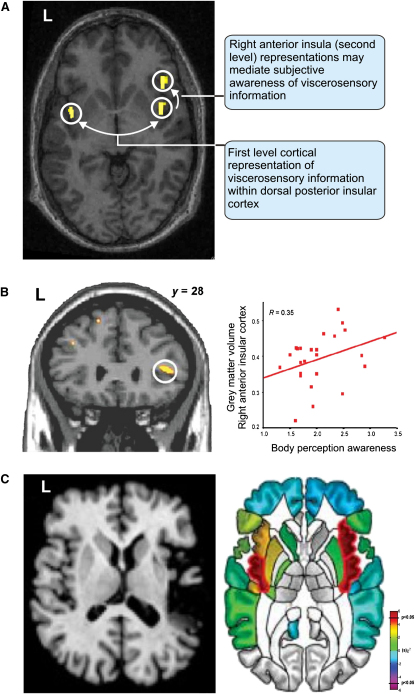
Interoception and Insula Cortex Activity (A) Bilateral projection fields within dorsal posterior insula cortex provide a cortical image of the physiological condition of the body. Second-level representations within the right anterior insula underlie the integration of interoceptive information with ongoing cognitive processing. (B) Increased interoceptive awareness is associated with increased functional activity within the right anterior insula. In addition, gray matter volume within this region predicts interoceptive awareness across subjects (see Critchley [2005]). (C) Patients with damage to the bilateral posterior and right anterior insula cortex were more likely to undergo a disruption of smoking addiction. Figure 2C reproduced from *Science*, Naqvi et al. (2007). Reprinted with permission from AAAS.

In our own laboratory, the link between interoception and insula activity has been observed in a number of contexts: including covariation of right anterior insula activity with sympathetic electrodermal activity during gambling or, in studies of patients with peripheral autonomic neuropathy, an attenuation of right insula activity in the absence of autonomic reaction during effortful behavior or during the processing of threat (reviewed in Critchley [2005]). In the latter study, regions of mid- and anterior insula were implicated integrating the interoceptive representation of threat-related bodily arousal responses with conscious awareness of the threat. This observation is consistent with *interpretative* models of emotion where the actual emotion that is experienced arises from interpretation of the perceived context in which bodily arousal changed (Schachter and Singer, 1962; Wiens, 2005). In a further study, it was observed that, even in the absence of “arousal,” insula cortex activity is enhanced by conscious awareness of interoceptive signals such as heartbeats and in so doing predicts individual differences in day-to-day feelings of anxiety (Figure 2). The study of Naqvi and colleagues (2007) reports disruption of smoking addiction when patients sustained a lesion within left or right dorsal posterior insula or right anterior insula. Previous neuroimaging studies suggest the experience of craving may be associated with activity changes in insula regions supporting both first-level (Kilts et al., 2004) and second-level (Kilts et al., 2001) interoceptive representations. Naqvi et al. (2007) provide evidence which link feelings of craving within smokers to the interoceptive function of insula cortex.

In addition to insula cortex, anterior cingulate and orbitofrontal cortices are also anatomically and functionally associated with integrated autonomic control and are sensitive to interoceptive signals (Critchley, 2005). Damage to either orbitofrontal cortex or anterior cingulate regions can attenuate autonomic responsively to motivational cues and has been associated with changes in motivational and emotional behavior (Bechara, 2004). Naqvi and colleagues (2007) tested for (but did not find) an association between orbitofrontal damage and disruption of smoking addiction, suggestive of a functional anatomical dissociation of the processes involved in inhibiting habitual behavior and representation of feelings such as craving. It is not clear if the study of Naqvi and colleagues (2007) had sufficient power to examine the effects of anterior cingulate lesions, but it is interesting to note that surgical lesions to anterior cingulate as a treatment for pain produce an attenuation of autonomic and other symptoms of opiate drug withdrawal.

A number of laboratories, including our own, are attempting to dissect the neural mechanisms that support and mediate interactions between conscious perception, interoceptive control, and emotional feelings. In parallel, studies of the functional neurobiology of the cognitive control of anxiety and the facilitation of motivational “unlearning” and extinction highlight the importance of affective neuroscience to clinical therapeutics. In the study by Naqvi and coworkers, the effects of insula lesions on smoking addiction are reminiscent of an “interoceptive agnosia” arising from disruption of viscerosensory representations (Nauta, 1971; Damasio, 1994). Their study identifies a crucial neural substrate for craving and emphasizes the critical role of conscious feeling states in driving habitual behavior. Underpinning this role of insula cortex in craving is its status as viscerosensory cortex and as the neural substrate for interoceptive feeling states.

Why did not all smokers with insula lesions quit? Obviously a number of factors play a role in the maintenance of addiction, only some of which are usefully explained at the neural level. Of these, alterations in striatal dopaminergic reward-related processing are well recognized to play a critical role. The degree to which reward/goal-selection processes and interoceptive awareness of craving differentially contribute to drug-seeking behavior is not known. One important next step in further understanding the interplay between the multiple neural systems mediating addiction (Hyman et al., 2006) would be to dissociate striatal dopaminergic error-prediction signals (Pessiglione et al., 2006) from linear associations between subjective craving and right anterior insula activity within addicted patients.
